# Identification of a novel immune checkpoint molecule V-set immunoglobulin domain-containing 4 that leads to impaired immunity infiltration in pancreatic ductal adenocarcinoma

**DOI:** 10.1007/s00262-023-03438-y

**Published:** 2023-04-25

**Authors:** Yongsheng Jiang, Lijie Han, Jian Yang, Minwei Yang, Jian Zhang, Meilin Xue, Youwei Zhu, Cheng Xiong, Minmin Shi, Shiwei Zhao, Baiyong Shen, Zhiwei Xu, Lingxi Jiang, Hao Chen

**Affiliations:** 1 Department of General Surgery, Pancreatic Disease Center, Ruijin Hospital, Shanghai Jiaotong University School of Medicine, Shanghai, China; 2 Research Institute of Pancreatic Diseases, Shanghai Jiaotong University School of Medicine, Shanghai, China; 3 State Key Laboratory of Oncogenes and Related Genes, Shanghai, China; 4 Department of Biliary-Pancreatic Surgery, Ren Ji Hospital, School of Medicine, Shanghai Jiao Tong University, Shanghai, People’s Republic of China; 5 Medical Department Health Services Section, Qingdao Women and Children’s Hospital, Qingdao, People’s Republic of China; 6 Institute of Translational Medicine, Shanghai Jiaotong University, Shanghai, China

**Keywords:** VSIG4, Pancreatic cancer, Immunity, T cells, Biomarkers

## Abstract

**Background:**

Checkpoint-based immunotherapy has failed to elicit responses in the majority of patients with pancreatic cancer. In our study, we aimed to identify the role of a novel immune checkpoint molecule V-set Ig domain-containing 4 (VSIG4) in pancreatic ductal adenocarcinoma (PDAC).

**Methods:**

Online datasets and tissue microarray (TMA) were utilized to analyze the expression level of VSIG4 and its correlation with clinical parameters in PDAC. CCK8, transwell assay and wound healing assay were applied to explore the function of VSIG4 in vitro. Subcutaneous, orthotopic xenograft and liver metastasis model was established to explore the function of VSIG4 in vivo. TMA analysis and chemotaxis assay were conducted to uncover the effect of VSIG4 on immune infiltration. Histone acetyltransferase (HAT) inhibitors and si-RNA were applied to investigate factors that regulate the expression of VSIG4.

**Results:**

Both mRNA and protein levels of VSIG4 were higher in PDAC than normal pancreas in TCGA, GEO, HPA datasets and our TMA. VSIG4 showed positive correlations with tumor size, T classification and liver metastasis. Patients with higher VSIG4 expression were related to poorer prognosis. VSIG4 knockdown impaired the proliferation and migration ability of pancreatic cancer cells both in vitro and in vivo. Bioinformatics study showed positive correlation between VSIG4 and infiltration of neutrophil and tumor-associated macrophages (TAMs) in PDAC, and it inhibited the secretion of cytokines. According to our TMA panel, high expression of VSIG4 was correlated with fewer infiltration of CD8^+^ T cells. Chemotaxis assay also showed knockdown of VSIG4 increased the recruitment of total T cells and CD8^+^ T cells. HAT inhibitors and knockdown of STAT1 led to decreased expression of VSIG4.

**Conclusions:**

Our data indicate that VSIG4 contributes to cell proliferation, migration and resistance to immune attack, thus identified as a promising target for PDAC treatment with good prognostic value.

## Background

PDAC is the most aggressive tumor with only 8% of 5-year survival rate [1]. Classically, PDAC exhibits an immunologically “cold” tumor microenvironment (TME) characterized by a prominent myeloid cell infiltration, limited CD8^+^ T (Teffector) cells infiltration and low activation marker expression (e.g., IFNγ). That means abnormal adaptive T cell immunity and resistance to checkpoint blockade [2]. Although newly developed targeted small molecules, chemotherapy combined with immunotherapies, have achieved great advance in various tumor types, the use of immune checkpoint (IC) inhibitors for the treatment of PDAC still shows limited effect [3]. Therefore, it is imperative to find more effective and potential biomarkers to facilitate novel therapeutic methods.

In addition to the detailed investigation of PD-1/PDL-1 and CTLA4 in tumor immunotherapies, recent studies have also focused on new IC targets, including LAG-3, TIM-3 and TIGIT, which form the second tier of IC molecules with more distinct and specific roles in immune response regulation [4]. Multiple clinical research has shown great potential in combination of newly developed IC inhibitor with traditional one. The combination of relatlimab, a LAG-3-blocking antibody, and nivolumab, a PD-1-blocking antibody, has shown to be safe and more powerful than nivolumab alone in patients with previously treated or untreated advanced melanoma (median PFS 10.1 months vs 4.6 months, HR = 0.75 and *P* = 0.006) [5]. A phase I/Ib clinical trial also showed that sabatolimab, an anti-TIM-3 antibody in combination of anti-PD-1 antibody, exhibited preliminary signs of anti-tumor activity, which provide a new therapeutic plan for PD-1 treatment-unresponsive cohort [6]. Besides, anti-TIGIT mAb (tiragolumab) has demonstrated promising clinical efficacy in non-small cell lung cancer treatment when combined with an anti-PD-L1 drug (Tecentriq), leading to phase III trial initiation [7].

VSIG4, normally expressed on subsets of tissue-resident macrophages, is a component of complement receptor of the immunoglobulin superfamily (CRIg) that belongs to the B7-related family [8]. VSIG4 is a second-tier immune checkpoint molecule that functionally inhibits the production of IL-2 and the proliferation of effector CD8^+^ T cells and induces regulatory T cells (Tregs) [9]. Administration to mice of soluble VSIG4-Ig fusion molecules reduced the induction of T cell responses in vivo [9]. The VSIG4^+^ macrophages seem to protect against the development of islet inflammation and *β* cell abnormalities in obesity, alleviate alcoholic liver disease, promote the clearance of blood-borne parasites and keep the homeostasis of Kupffer cells [10–14]. VSIG4 also inhibit the activation of pro-inflammatory macrophages by reprogramming mitochondrial pyruvate metabolism [15]. However, since VSIG4 is characterized as a macrophage-specific molecule, neither the expression nor function of VSIG4 in PDAC has been reported yet.

Considering its potential role in immunotherapy, delineation of the prognostic value and its molecular function in immune system is potent. Therefore, we evaluated the biological function of VSIG4 and its prognostic impact in PDAC. In addition, its function in remodeling immune microenvironment was also investigated.

## Methods

### Patient datasets

To perform pan-cancer analysis of VSIG4, the mRNA expression level of patients with corresponding clinical parameters was extracted from TCGA ((https://cancergenome.nih.gov). Expression level of corresponding normal tissues was extracted from GTEx dataset (www.gtexportal.org/). GEO datasets including GSE15471, GSE102238 and GSE15932 were downloaded from GEO database (https://www.ncbi.nlm.nih.gov/geo/). The protein expression levels of VSIG4 in pancreatic cancer tissue and normal pancreas were extracted from The Human Protein Atlas (HPA) database (http://www.proteinatlas.org/).

### Immunohistochemistry (IHC) staining

Studies using human tissues were reviewed and approved by the Committees for Ethical Review of Research involving Human Subjects of Renji Hospital affiliated with Shanghai Jiaotong University School of Medicine. Informed consents were obtained from all patients before study inclusion. Immunohistochemistry protein expression was determined in PDAC cells from archived formalin-fixed paraffin-embedded (FFPE) tumors from patients who underwent surgical treatment at Renji Hospital. Tumor tissues were scored according to the percentage of stained area (0 = 0%–5%, 1 = 6%–35%, 2 = 36%–70%, and 3 = more than 70%) and intensity of the nuclei or cytoplasm staining (0 OD value = 0, 0–1 OD value = 1, 1–2 OD value = 2, 2 < OD value = 3, and mean OD value were measured by ImageJ). Final scores were determined by multiplying the two numbers mentioned above (“negative/−” for a score of 0–1, “weak/ + ” for a score of 2–3, “moderate/ +  + ” for a score of 4–6 and “strong/ +  +  + ” for a score of more than 6). Samples with scores higher than 3 were defined as high expression, and samples with no more than 3 were defined as having low expression. Antibody used in our work includes VSIG4 (ab252933, Abcam), CD8A (ab17147, Abcam), T-bet (sc-21763, Santa Cruz), GATA3 (ab199428, Abcam) and FOXP3 (22228-1-AP, ProteinTech). IHC of CD8A, T-bet, GATA3 and FOXP3 was performed in TMA consisting of 208 cases. IHC of VSIG4 was performed in TMA consisting of 208 plus 103 newly enrolled cases. The detailed procedure was previously described [16].

### Tissue microarray (TMA) construction

Detailed TMA construction procedure was previously described [16].

### Survival Analysis of tissue microarray (TMA)

Overall survival curves were calculated using the Kaplan–Meier method and were analyzed using the log-rank test by SPSS 17.0 (SPSS, Chicago, IL, USA).

### Cell lines and transfection

Cell lines AsPC-1 and Capan1 were obtained from Cell Bank of the Chinese Academy of Sciences. All cell lines were maintained at 37 °C and 5% CO2. AsPC-1 cells were cultured in DMEM (Meilunbio, China) containing 10% fetal bovine serum (FBS) (Gibco; Life Technology) and 50 μg/mL penicillin/streptomycin (P/S). CAPAN1 cells were cultured in IMDM (BIOAGRIO) containing 20% FBS (Gibco; Life Technology) and 50 μg/mL penicillin/streptomycin (P/S). To interfere the expression of VSIG4 and STAT1 in cell lines, RNAiMAX reagent (Thermo Fisher, USA) mixed with small interference RNA targeting VSIG4 was applied according to the manufacturer’s instructions. Short hairpin RNAs specifically targeting VSIG4 cell lines were generated using lentiviral vectors. A total of 300,000 PDAC cells were infected with viral supernatants and 10 μg/mL polybrene (Yeasen) for 6 h. Transfected cells were selected with 10 μg/mL puromycin to produce stable cell lines.

### Quantitative real-time PCR and Western blot (WB)

Detailed RNA extraction, quantitative real-time PCR and Western blot were previously described. Primers used were VSIG4 (forward: 5′-GGGGCACCTAACAGTGGAC-3′, reverse: 5′-GTCTGAGCCACGTTGTACCAG-3′) and 18S (forward: 5′-TGCGAGTACTCAACACCAAC-A-3′, reverse: 5′- GCATATCTTCGGCCCACA -3′). Antibodies used were VSIG4 (1:1000, ab252933, Abcam), E-cadherin (1:1000, GB14076, Servicebio), N-cadherin (1:1000, GB11135, Servicebio) and GAPDH (1:1000, 5174, Cell Signaling Technology).

### Cell proliferation assays

Cell Counting Kit-8 (CCK-8) assays were performed to determine the proliferation of cells. Procedure of detailed CCK8 assay was previously described [17].

### Cell migrate assay

Procedure of transwell and wound healing assay was previously described [18].

### Subcutaneous xenograft model, orthotopic xenograft and liver metastasis model

Subcutaneous implant models were established by subcutaneous injection at a total cell number of 5 × 10^6^ in 100 μl PBS. The mice were killed after 30 days. The subcutaneous tumors were isolated and weighed. Orthotopic xenograft models were utilized as reported previously [19]. For liver metastasis model, AsPC-1 cells were transfected with luciferase for bioluminescence imaging. A total cell number of 5 × 10^6^ in 25 μl PBS were injected into the spleen of mice. The mice were killed after 30 days.

#### Chemotaxis assay

Chemotaxis assay was performed as previously described [16].

#### Functional enrichment analysis and protein–protein interaction (PPI) network analysis

To perform functional enrichment analysis of VSIG4, top 50 most positively and top 50 negatively correlated genes with VSIG4 in PDAC were obtained based on TCGA database. These genes were enriched by Gene Ontology (GO) [including biological processes (BP), cellular components (CC) and molecular function (MF)] and KEGG pathway analyses using the Database for Annotation, Visualization and Integrated Discover (DAVID) and visualized by the R package ggplot2. STRING database (http://string-db.org) was selected to construct PPI network of VSIG4-related molecules.

#### Hub gene analysis

To identify the hub genes in the network, we analyzed the clusters of the network with several criteria (degree cut-off: 2; k-core: 2; node score cut-off: 0.2; and max depth: 100). Then, we calculated the node scores using the cytoHubba plug-in (version 0.1) in cytoscape. Top ten ranked nodes were selected as the hub genes of VSIG4.

#### Gene set enrichment analysis (GSEA)

Gene set enrichment analysis (GSEA) was performed as previously described [16].

#### Statistical analysis

Statistical analyses were performed using GraphPad Prism version 8.0. Cox regression analyses, and *χ*^2^ tests were used to assess the association with overall survival using SPSS v23 (IBM Inc.). An independent Student’s *t* test was used to compare the mean expression level of two different groups, and one-way analysis of variance was used to compare means between three or more subgroups. All statistical tests were two-tailed, with statistical significance indicated at *P* < 0.05. Pearson’s *χ*^2^ test was used to analyze the clinical pathological features and correlation of gene expression.

## Results

### VSIG4 is highly expressed in PDAC

**Fig. 1:**
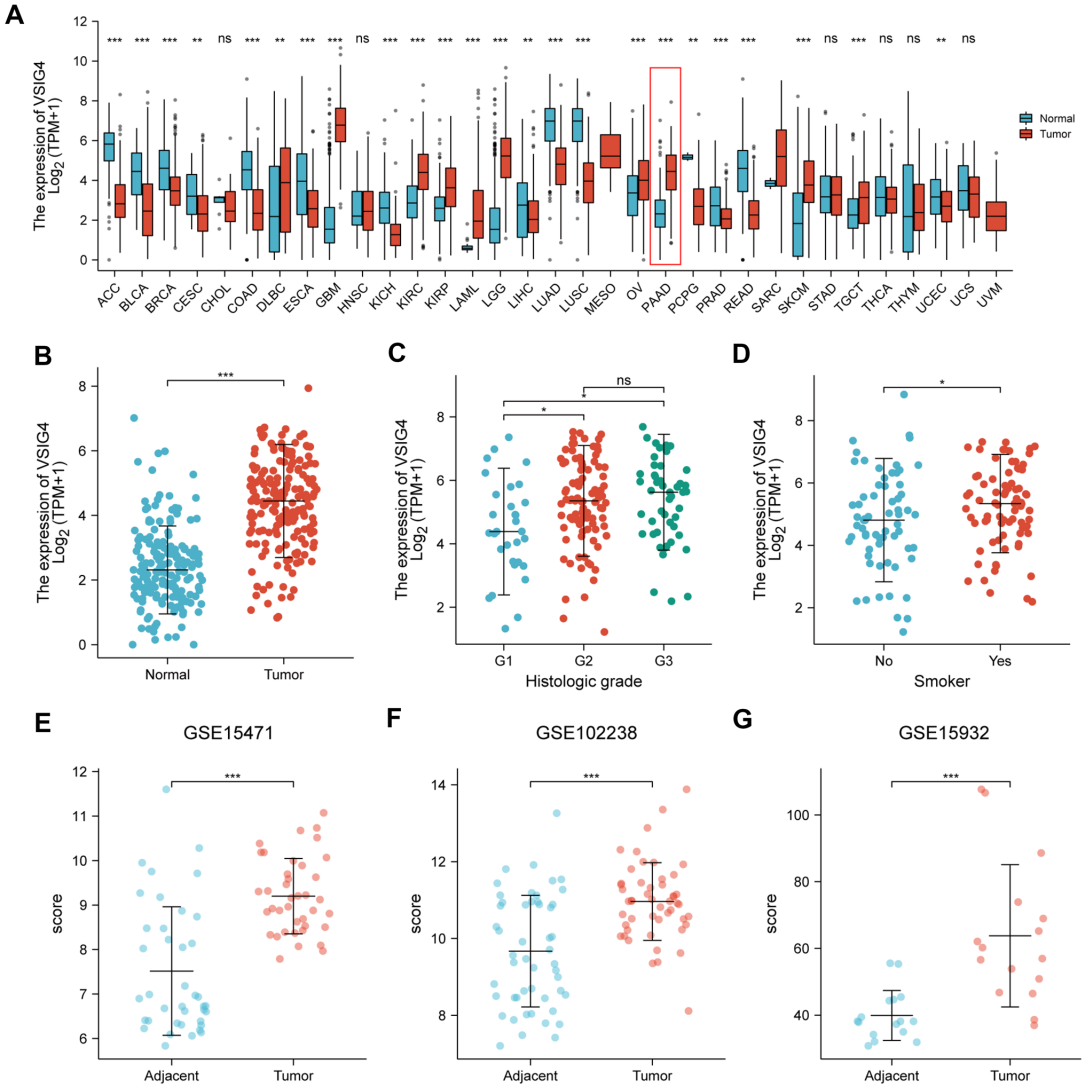
The expression level of VSIG4 in pan-cancer including PDAC. **A** mRNA expression levels of VSIG4 in 33 cancer types and corresponding normal tissue. **B** Expression of VSIG4 in samples of PDAC and normal pancreas in TCGA. Expression of VSIG4 in TCGA PDAC samples grouped by histological grade (**C**) or smoking history (**D**). Expression of VSIG4 in three GEO datasets GSE 15,471 (**E**), GSE102238 (**F**) and GSE 15,932 (**G**) **H** Protein expression level of VSIG4 in PDAC and normal pancreas in HPA. Bar graphs are presented as mean + SD (Unpaired two-tailed Student *t* test; *P < 0.05, **P < 0.01, ***P < 0.001 and ****P < 0.0001)

By datamining in TCGA and GTEx datasets, the expression pattern of VSIG4 in 33 cancer types and corresponding normal tissues was determined. We found that the expression level of VSIG4 was various in different cancer types (Fig. 1A). Among them, VSIG4 is evidently highly expressed in PDAC, which is the most prevalent primary pancreatic cancer, than normal pancreas (Fig. 1B). Moreover, clinical features including histologic grade and smoking history validated as a risk factor of PDAC are associated with VSIG4 expression (Fig. 1C, D). To further investigate the expression of VSIG4 in pancreatic cancer, three datasets from GEO database were selected. As seen in Fig. 1E–G, expressions of VSIG4 were consistently higher in tumor tissues than adjacent normal pancreas in all three datasets. These data suggested that according to several independent external datasets, VSIG4 was significantly upregulated in pancreatic cancer.

### Validation using tissue microarray

**Fig. 2:**
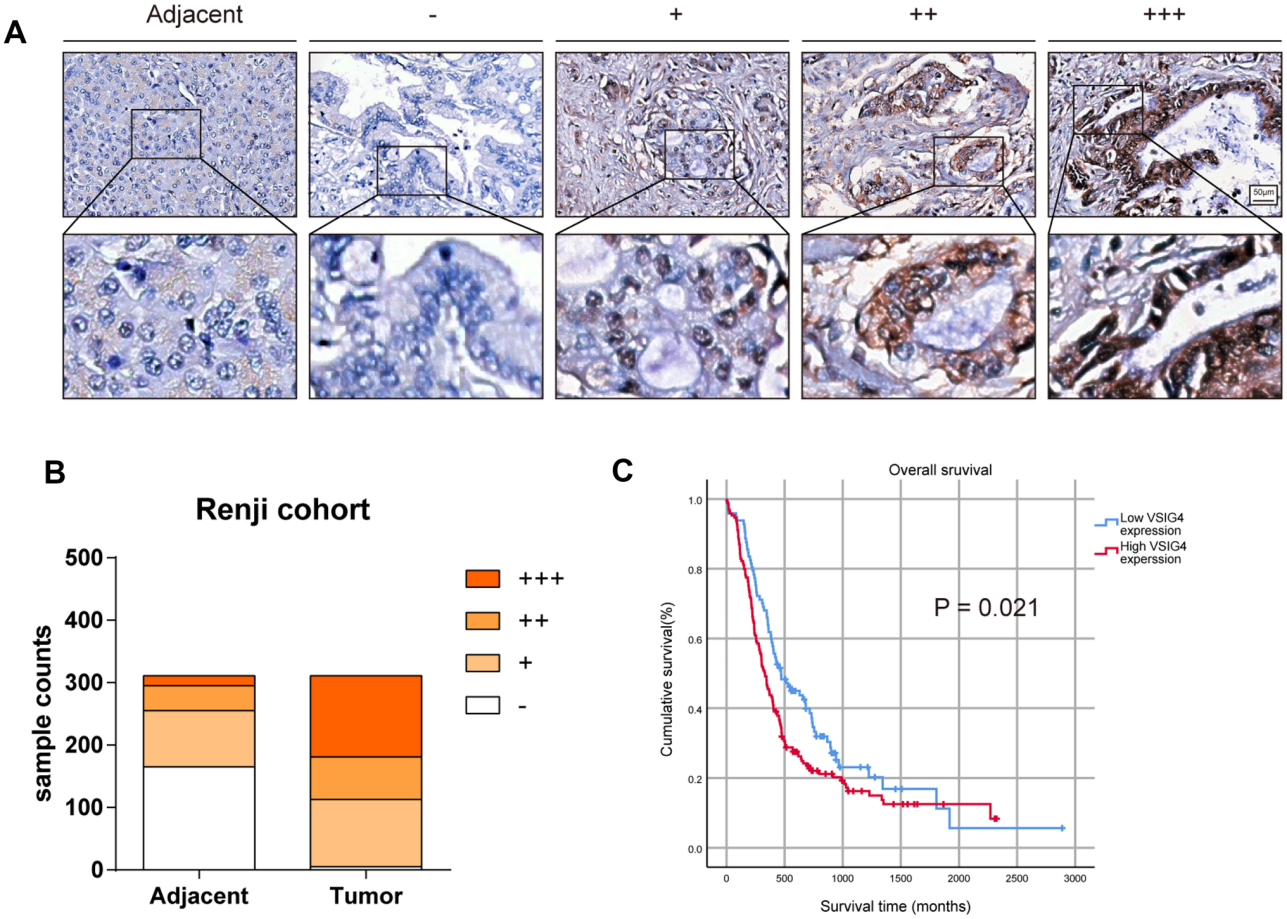
Expression pattern of VSIG4 in Renji TMA. **A** Representative IHC images of VSIG4 expression in Renji TMA spotted with human PDAC tissue cores. **B** Respective sample counts in tumor tissues and adjacent tissues. **C** Patients with high VSIG4 expression accompanied with poor prognosis. Bar graphs are presented as mean + SD (unpaired two-tailed Student *t* test; *P < 0.05, **P < 0.01, ***P < 0.001 and ****P < 0.0001)

After determining the expression level of VSIG4 in external dataset, we analyzed the protein expression level of VSIG4 in our TMA containing 311 paired PDAC tissues and corresponding adjacent pancreas. Samples were scored according to both percentage of stained area and intensity and determined as negative, weak, moderate and strong. As shown in Fig. 2A, VSIG4 was specifically expressed in cytoplasm of tumor cells. Consistent with result in HPA database, the expression level of VSIG4 is significantly higher in tumor tissues than that in normal pancreas (Fig. 2B). To further investigate whether the expression of VISG4 is associated with patient survival, 311 PDAC patients were divided into high VSIG4 expression group (samples scored moderate or strong) and low VSIG4 expression group (samples scored negative or weak). By performing Kaplan–Meier survival analysis, we found that patients with higher VSIG4 expression were related to poorer prognosis (Fig. 2C). To further elucidate the correlation between VSIG4 and clinical characteristics in PDAC patients, we then performed Chi-square analysis in TMA cohort. As shown in Table 1, characteristics including age, gender, tumor location, size, tumor differentiation, T classification, liver metastasis, vascular invasion and neural invasion were analyzed. The result showed that high expression was associated with T classification and liver metastasis. We also performed univariate and multivariate analyses to determine the risk factors correlated with patient’s prognosis in TMA cohort (Table 2). According to univariate analysis, VSIG4 expression level, tumor size, T classification, liver metastasis and vascular invasion are risk factors of patients’ survival. Moreover, only tumor size and vascular invasion were risk factors according to multivariate analysis.

**Table 1:** Correlation between VSIG4 expression and clinicopathologic factors

Characteristics	Total	VSIG4 expression		*P* value (*χ*^2^ test)
		High (*n* = 198)	Low (*n* = 113)	
*Age (years)*				0.957
< 60	93	59	34	
≥ 60	218	139	79	
*Gender*				0.990
Male	176	112	64	
Female	135	86	49	
*Tumor location*				0.199
Head	206	126	80	
Body/tail	105	72	33	
*Size*				0.193
≤ 3 cm	112	66	46	
> 3 cm	199	132	67	
*Tumor differentiation*				0.669
Well	17	10	7	
Moderate/poor	294	188	106	
*T classification*				0.000*
T1,2	243	134	109	
T3,4	68	64	4	
*Liver metastasis*				0.000*
Absent	281	168	113	
Present	30	30	0	
*Vascular invasion*				0.093
Absent	280	174	106	
Present	31	24	7	
*Neural invasion*				0.471
Dead	165	102	63	
Alive	146	96	50	

*P < 0.05

**Table 2:** Univariate and multivariate Cox regression analysis of potential prognostic factors in pancreatic cancer

Prognostic parameter	Univariate analysis			Multivariate analysis		
	RR	95%CI	*P* value	RR	95%CI	*P* value
VSIG4 (low vs. high)	1.395	1.049–1.855	0.021*	1.140	0.833–1.559	0.412
Age (< 60 vs. ≥ 60)	1.187	0.875–1.610	0.271			
Gender (male vs. female)	0.861	0.655–1.132	0.283			
Tumor location (head vs. body/tail)	1.039	0.782–1.380	0.791			
Size (≤ 3 cm vs. > 3 cm)	1.753	1.313–2.341	0.000*	1.524	1.126–2.063	0.006*
Tumor differentiation (well vs. moderate/poor)	1.242	0.657–2.349	0.505			
T classification (T1 vs. T2 vs. T3 vs. T4)	1.827	1.326–2.518	0.000*	1.544	0.994–2.398	0.053
Liver metastasis (absent vs. present)	1.858	1.201–2.874	0.005*	1.119	0.640–1.958	0.692
Neural invasion (absent vs. present)	1.147	0.876–1.502	0.319			
Vascular invasion (absent vs. present)	0.005	1.195–2.802	0.005*	1.727	1.098–2.717	0.018*

*P < 0.05

### VSIG4 contributes to tumor proliferation and migration

**Fig. 3:**
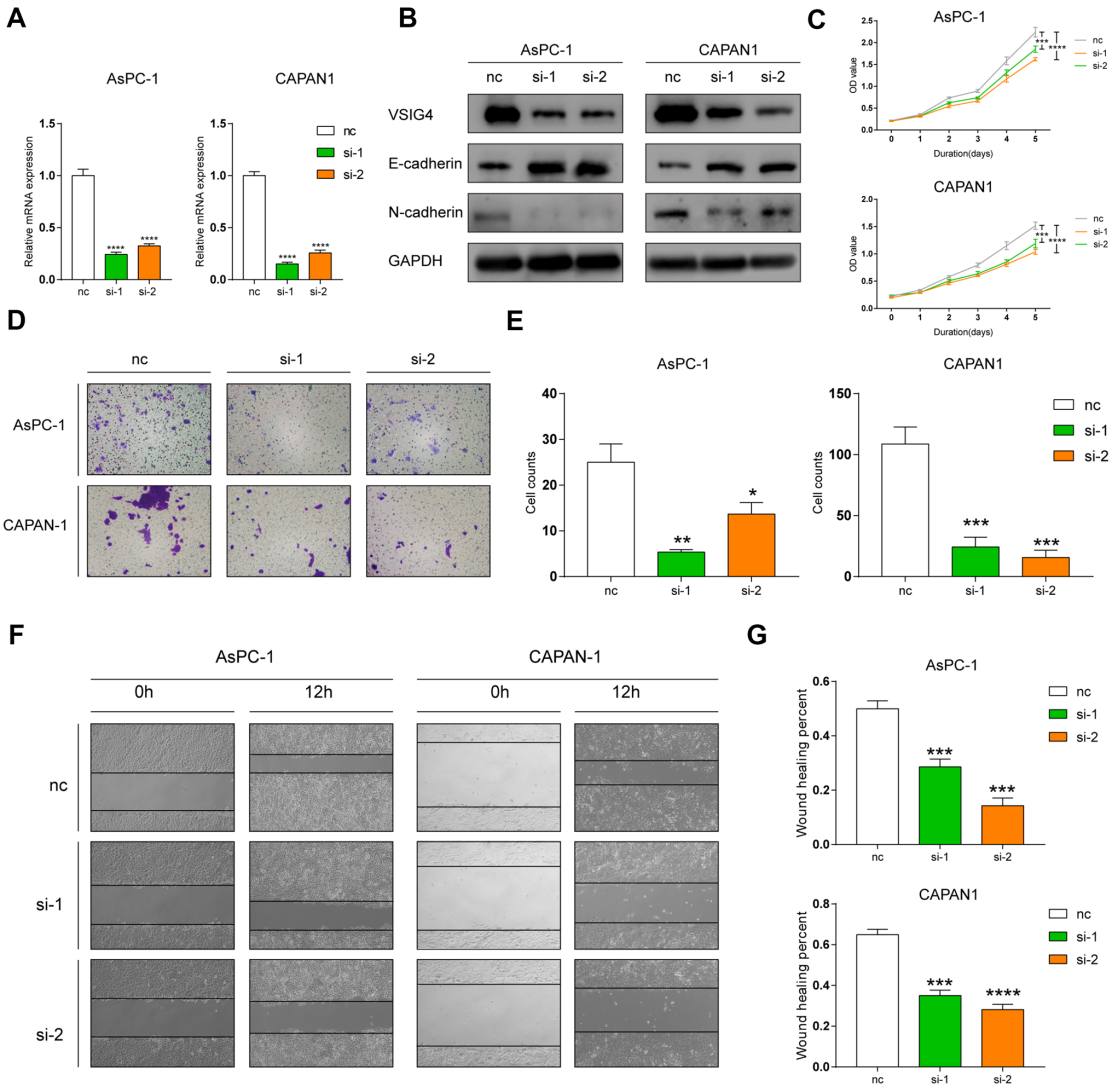
VSIG4 contributes to proliferation and migration of PDAC cells in vitro. Knockdown efficiency of si-RNA targeting VSIG4 in RNA (**A**) and protein levels (**B**). **C** Cell proliferation of pancreatic cancer cells were measured by CCK8. **D & E** Cell migration of pancreatic cancer cells were measured by transwell assay. **F & G** Cell migration of pancreatic cancer cells were measured by wound healing assay. Bar graphs are presented as mean + SD (*n* = 3; unpaired two-tailed Student *t* test; *P < 0.05, **P < 0.01, ***P < 0.001 and ****P < 0.0001)

As high expression of VSIG4 was validated in PDAC tissue and negatively correlated with PDAC patients’ prognosis, we then intended to explore its biological function in pancreatic cancer cells. VSIG4-targeted si-RNA sequences were selected to knockdown VSIG4 in cells. As shown in Fig. 3A and B, both mRNA and protein expression levels of VSIG4 in two pancreatic cancer cell lines were significantly decreased. We then performed CCK-8 assay to detect the impact of VSIG4 on cell proliferation. As shown in Fig. 3C, knockdown of VSIG4 evidently impaired the proliferation of both AsPC-1 and CAPAN1 cell lines. The effect of VSIG4 on cell migration was also investigated. Transwell assay was firstly applied. The result showed that fewer cells with VSIG4 knockdown migrated to the downside of the chamber compared with control group (Fig. 3D and E). Consistently, when VSIG4 is silenced, cells migration area was also smaller than the control group in the wound healing assay (Fig. 3F and G). Also, epithelial mesenchymal transition (EMT) markers were also detected, indicating that VSIG4 contributes to EMT process in pancreatic tumor cells (Fig. 3B).

**Fig. 4:**
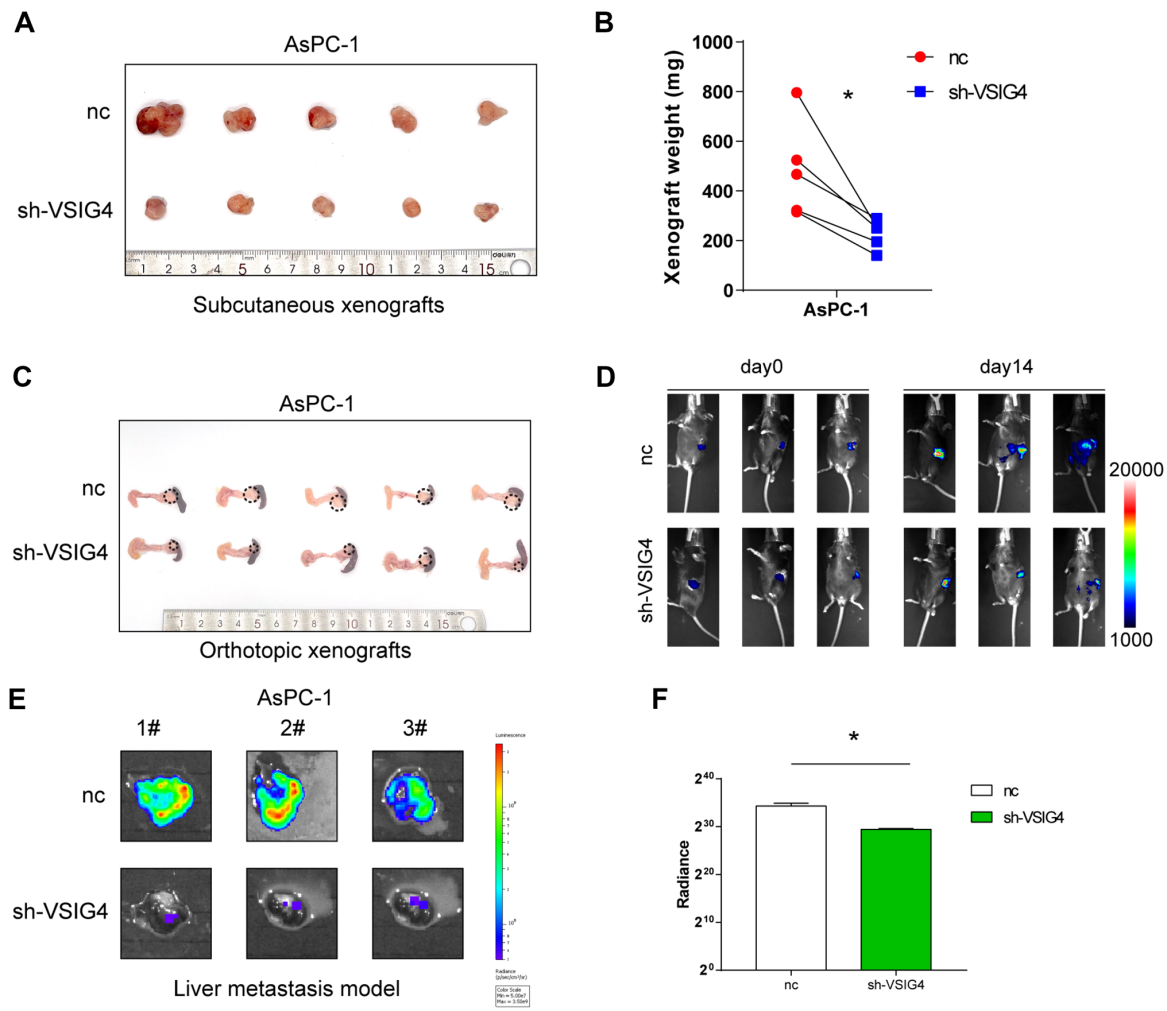
VSIG4 contributes to proliferation and invasion of PDAC cells in vivo. **A** & **B** The negative control and sh-VSIG4-1 AsPC-1 cells were injected subcutaneously in nude mice. Xenografts were collected 30 days after injection. **C** The negative control and sh-VSIG4-1 AsPC-1 cells were injected into pancreas body of nude mice. Pancreases were collected 30 days after injection. **D** The negative control and sh-VSIG4-1 luciferase-expression AsPC-1 cells were injected into the spleen of nude mice. Image of IVIS spectrum of liver was collected 30 days after injection. **E** The quantification of liver metastatic emission images. Bar graphs are presented as mean + SD (unpaired two-tailed Student *t* test; *P < 0.05, **P < 0.01, ***P < 0.001 and ****P < 0.0001)

Furthermore, to detect the effect of VSIG4 on PDAC in vivo, three animal models were used. For the subcutaneous xenograft model, the average volume of tumors induced by VSIG4 knockdown group was significantly smaller than that of the control group (Fig. 4A, B). Pancreatic orthotopic tumor formation model exhibited the same result (Fig. 4C). To better reflect the influence of VSIG4 on PDAC in the presence of T cells, we also performed orthotopic tumor formation model in C57 mice using Panc02 cell line. The result showed that knockdown of VSIG4 significantly impairs the proliferation of PDAC cells in C57 pancreas (Fig. 4D). Liver metastasis models also showed that knockdown of VSIG4 evidently impaired the metastasis of PDAC cells to liver (Fig. 4E, F). These results suggested that VSIG4 facilitates tumor proliferation and migration to contribute to its progression.

### Identification of differential and hub genes correlated with VSIG4 in PDAC

**Fig. 5:**
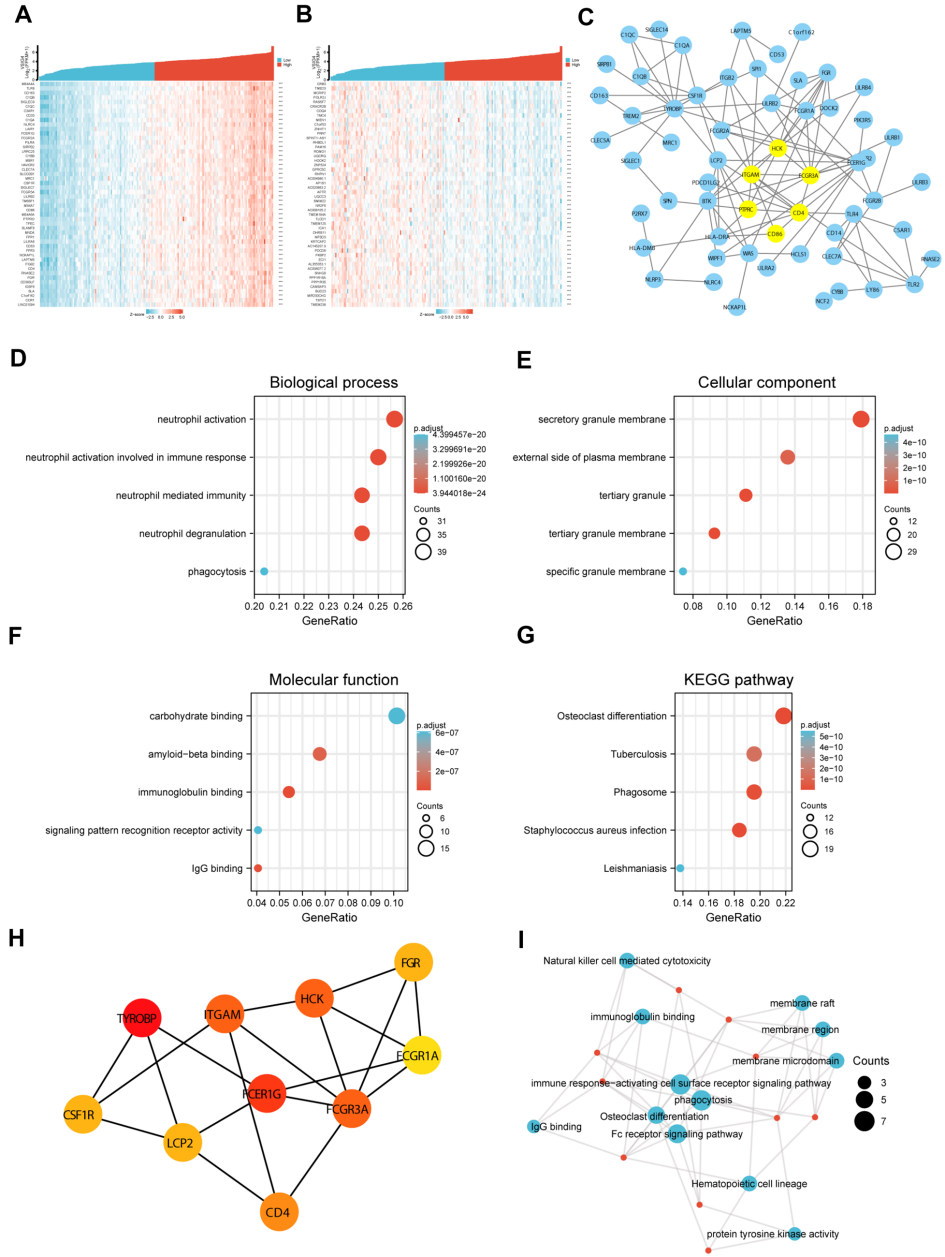
VSIG4 functional clustering and interaction network analysis of VSIG4-related genes. **A & B** Heatmap of top 50 most positively correlated and top 50 negatively correlated genes of VSIG4 in PDAC according to TCAG. **C** VSIG4-related gene interaction network according to STRING. **D**–**G** GO and KEGG analysis revealed related pathways of the 50 most positively correlated genes of VSIG4. **H** The interaction network of top ten hub genes related to VSIG4. I. GO and KEGG analysis of the hub genes

We then identified differentially expressed genes correlated with VSIG4 in PDAC, we applied Spearman test based on the TCGA database. The top 50 most positively and top 50 most negatively correlated genes of VSIG4 were shown in heatmap (Fig. 5A and B). Then, we selected top 170 most positively correlated genes with coefficient > 0.7 for further analysis. By uploading the 170 genes into STRING, the PPI network of them was constructed (Fig. 5C). We then performed GO and KEGG enrichment analysis. Consistently with previously reported, VSIG4-correlated genes were significantly enriched in immune-related pathways [20]. Affected biological process includes neutrophil activation, neutrophil-mediated immunity, neutrophil degranulation and phagocytosis (Fig. 5D). Involved cellular component includes secretory granule membrane, external side of plasma membrane, tertiary granule, etc. (Fig. 5E). Involved molecular function mainly enriched in carbohydrate binding and amyloid-beta binding immunoglobulin binding. (Fig. 5F). The KEGG analysis indicated that involved pathways mainly enriched in osteoclast differentiation, tuberculosis, phagosome, etc. (Fig. 5G).

To identify the hub genes of VSIG4 in PDAC, we firstly located the core clusters in the PPI network by utilizing MCODE (Fig. 5C, shown in yellow). The top ten hub genes were then identified by cytoHubba (Fig. 5H). GO and KEGG analysis of the hub genes also mostly enriched in immune-related pathways like phagocytosis and NK cell-mediated cytotoxicity. (Fig. 5I). These results indicated that VSIG4 may participated in the immune reaction during PDAC progression.

### Expression of VSIG4 negatively correlated with CD8^+^ T cells infiltration in PDAC

**Fig. 6:**
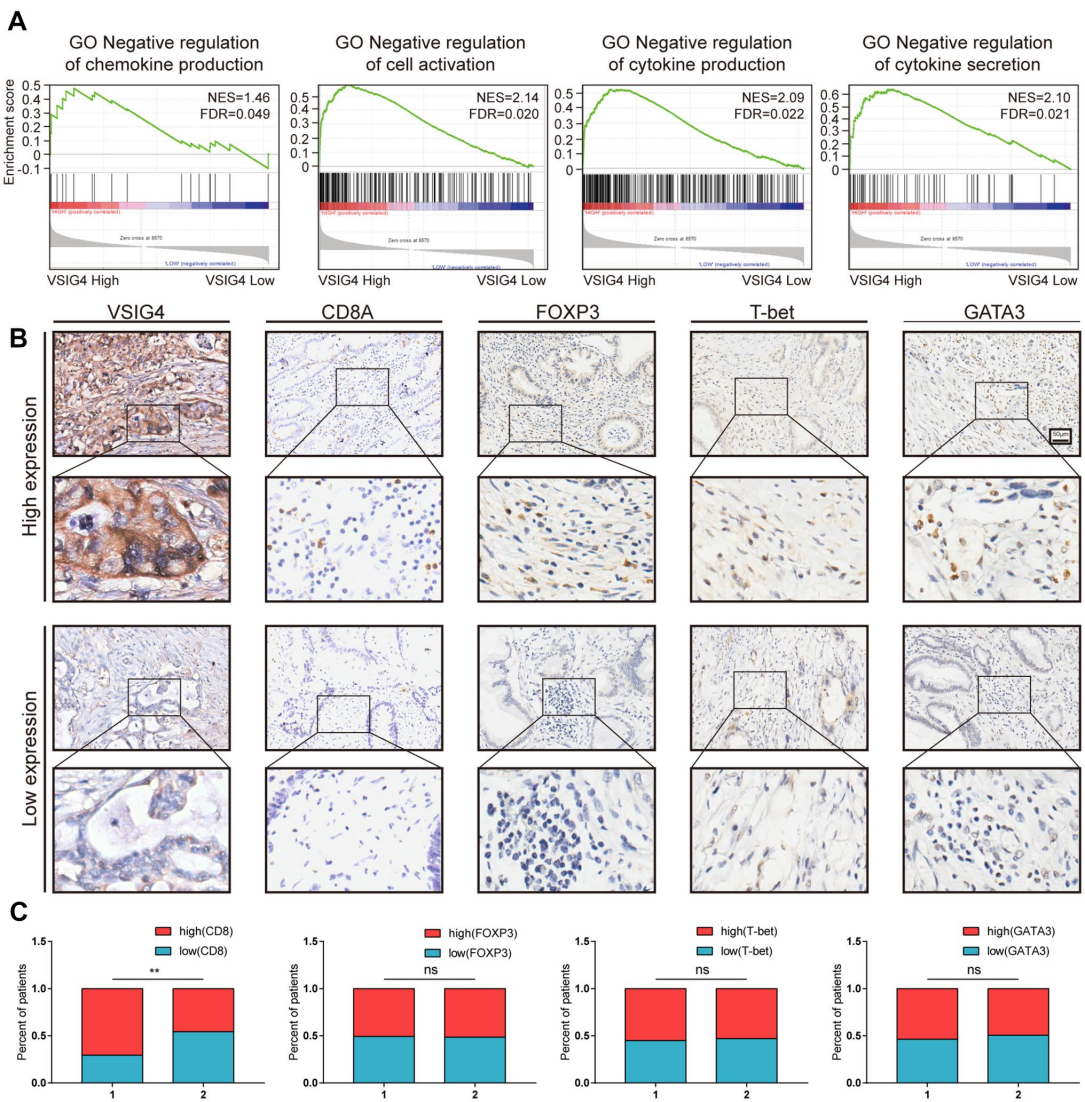
Correlation analysis of VSIG4 expression and immune infiltration in PDAC. **A**. GSEA analysis of VSIG4-related pathways according to TCGA. **B**. Representative IHC images of VSIG4, CD8A, FOXP3, T-bet and GATA3 expression in Renji TMA. **C**. Patients were divided into two groups according to the expression of VSIG4, and the column bar graphs showed the correlation between CD8A, FOXP3, T-bet, GATA3, and VSIG4. Bar graphs are presented as mean + SD (unpaired two-tailed Student *t* test; *P < 0.05, **P < 0.01, ***P < 0.001 and ****P < 0.0001)

**Fig. 7:**
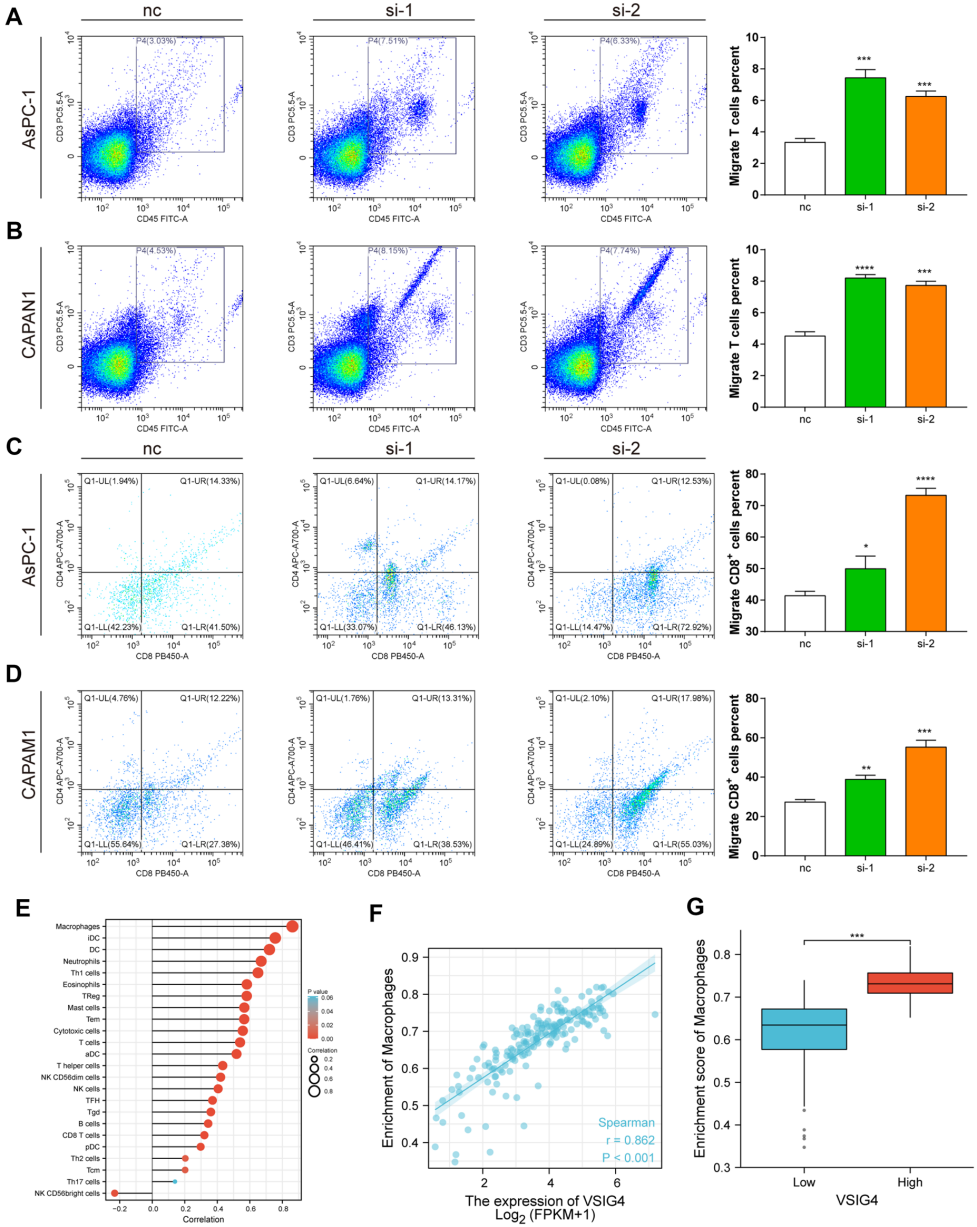
VSIG4 impairs the recruitment of CD8^+^ T cells and neutrophils. **A & B** Proportion of T cells of total migrated PBMCs counted by flow cytometry. **C & D** Proportion of CD8^+^ and CD4^+^ T cells of total migrated T cells counted by flow cytometry. **E** Infiltration of immune cells in patients with high VSIG4 expression and low VSIG4 expression. **F** Macrophages’ marker was positively correlated with VSIG4 in PDAC according to TCGA. **G** Enrichment score of macrophages in VSIG4 high and low group in PDAC according to TCGA. Bar graphs are presented as mean + SD (*n* = 3; unpaired two-tailed Student *t* test; *P < 0.05, **P < 0.01, ***P < 0.001 and ****P < 0.0001)

To further investigate the effect of VSIG4 on PDAC immune system, we firstly performed GSEA in TCGA according to VSIG4 expression. The result showed that high expression of VSIG4 was negatively correlated with several immune reaction like cytokine production and secretion (Fig. 6A). We then analyzed the correlation between VSIG4 and Treg, CD8^+^ T cells, Th2 and Th1 cell using established TMA panel as previously described[16] (Fig. 6B). As Fig. 5C shows, high expression of VSIG4 was significantly correlated with less infiltration of CD8^+^ T cells, while no significant difference was shown in other cell types (Fig. 6C). We then performed chemotaxis assay to confirm the effect of VSIG4 on immune cell recruitment. Culture medium of different tumor cell groups was added in the lower chamber to recruit peripheral blood mononuclear cell. After 2 h of recruitment, cells were collected and analyzed by flow cytometry. The results suggested that knockdown of VSIG4 led to the recruitment of total T cells (Fig. 7A, B). The proportion of CD8^+^ T cells were also studied. The result suggested that VSIG4 significantly decreased the percentage of migrated CD8^+^ T cells (Fig. 7C, D).

Furthermore, we analyzed the correlation between VSIG4 and 24 types of immune cells in PDAC via TIMER database (Fig. 7E). Consistent with previous study, VSIG4 is positively correlated with macrophage infiltration (Fig. 7F). The enrichment score of macrophage is significantly higher in VSIG4 high group in PDAC according to TCGA (Fig. 7G). These results indicated that VSIG4 may impair the recruitment of CD8^+^ T cells while facilitate the recruitment of macrophages during the progression of PDAC.

### H3K27 acetylation and hypoxia facilitate transcription of VSIG4

**Fig. 8:**
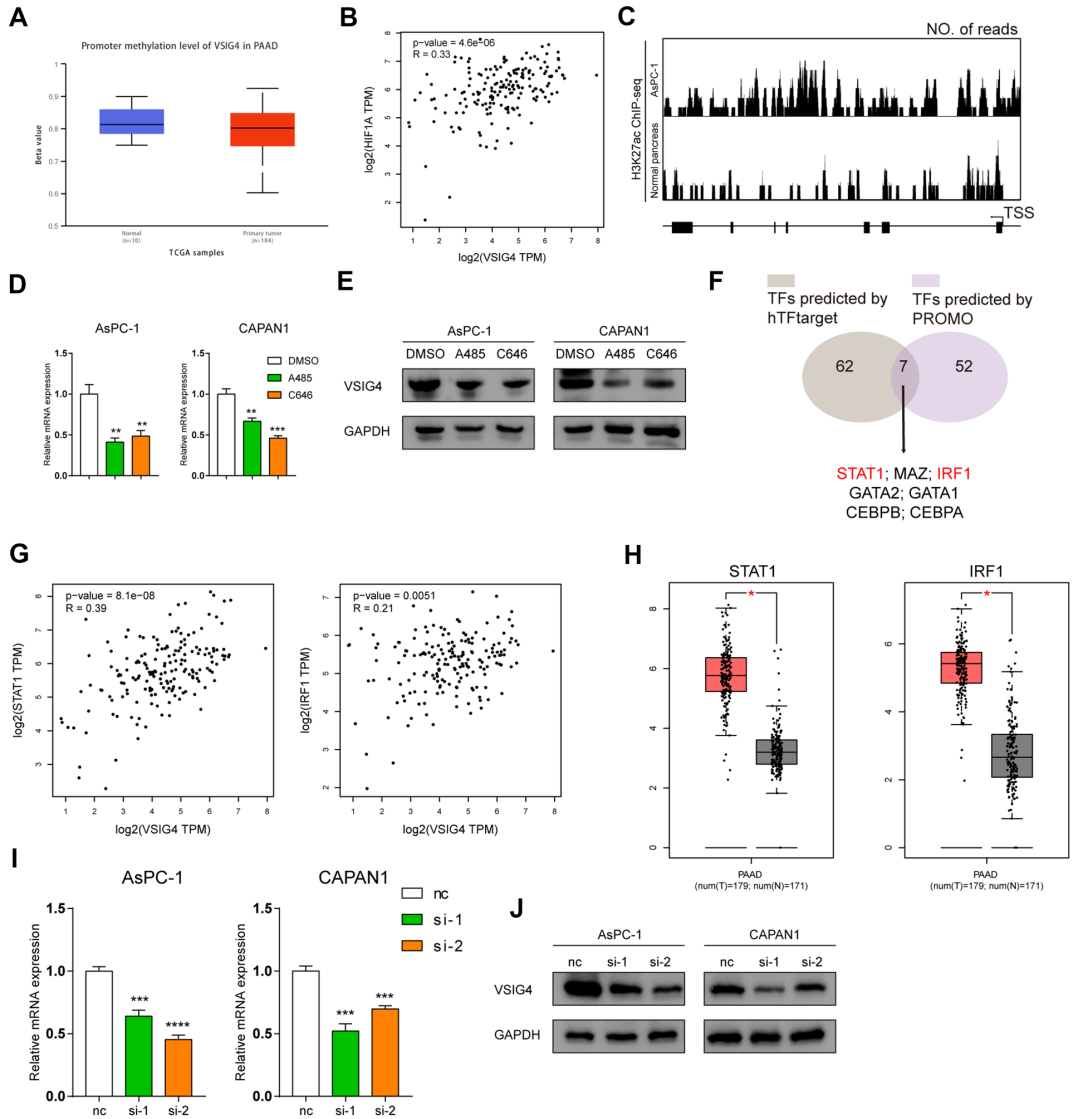
H3K27 acetylation and hypoxia facilitates transcription of VSIG4. **A** Promoter methylation level of VSIG4 in PDAC and normal pancreas. **B** Correlation between HIF1α and VSIG4 in PDAC. **C** Predicted H3K27ac binding site in VSIG4 promoter from UCSC genome browser. Real-time expression of VSIG4 in AsPC-1 and CAPAN1 treated with DMSO, A485 or C646 in RNA (**D**) and protein (**E**) levels. **F** Venn diagram showing TF screening strategy. **G** Both STAT1 and IRF1 are positively correlated with VSIG4 in PDAC according to TCGA. **H** Both STAT1 and IRF1 are upregulated in PDAC according to TCGA and GTEx. Real-time expression of VSIG4 in AsPC-1 and CAPAN1 transfected with si-STAT1 or negative control in RNA (**I**) and protein (**J**) levels. Bar graphs are presented as mean + SD (*n* = 3; unpaired two-tailed Student *t* test; *P < 0.05, **P < 0.01, ***P < 0.001 and ****P < 0.0001)

VSIG4 was identified as a macrophage-specific molecule before. How does it expressed in PDAC tissue also deserved being investigated. Since the epigenetic alterations, including DNA methylation, histone modifications and noncoding RNA expression, have been reported to play a major role in the gene regulation of cancer, we firstly compared the promoter methylation profile between PDAC and normal pancreas according to TCGA. However, no significant difference was observed (Fig. 8A). In consideration of hypoxia tumor microenvironment (TME) of PDAC, we then explored whether hypoxia could increase the expression of VSIG4. As shown in Fig. 8B, VSIG4 exhibited strong positive correlation with the master hypoxia transcription factor HIF1α. The hypoxia-associated mRNA expression patterns are mainly due to alterations of histone modification in PDAC. Among them, H3K27 acetylation (H3K27ac) is a well-established marker for transcription activation. To unveil the mechanism of VSIG4 upregulation in PDAC, the transcriptional modification of VSIG4 from the UCSC genome browser was investigated. As shown in Fig. 8C, compared with normal pancreas, H3K27ac binding peaks were evidently enriched in AsPC-1 cell lines in VSIG4 promoter region. Two histone acetyltransferase (HAT) inhibitors, A-485 and C646, were used to treat two PDAC cell line and significantly decreased the expression of VSIG4 both in mRNA and protein levels (Fig. 8D, E). To predict the potential transcription factor (TF) of VSIG4, we input upstream 2000bps of VSIG4 promoter region. Seven TFs were screened out, while STAT1, IRF1, GATA2, GATA1 and CEBPA exhibit positive correlation with VSIG4 in PDAC (Fig. 8F). Among them, only STAT1 and IRF1 were highly expressed in PDAC (Fig. 8G and H). Since STAT1 exhibited stronger correlation with VSIG4, we knocked down the expression of STAT1 in both cell lines that subsequently led to decreased expression of VSIG4 (Fig. 8I, J).Therefore, STAT1 were identified as the potential transcription factor of VSIG4 in PDAC.

## Discussion

Although surgery and adjuvant chemotherapy can achieve long-term survival, nearly all PDAC patients eventually relapse because of recurrence or liver metastasis [21]. Also, second-line options such as immune monotherapies are extremely poor and have little clinical activity in PDAC. Therefore, the identification of reliable target could lead to better outcomes and improve effect of immunotherapies in pancreatic cancer. This study is the first to evaluate VSIG4 expression and its influence on immune microenvironment in PDAC. We found that VSIG4 expression in PDAC is strongly associated with poor OS in a local cohort and its expression value is positive correlated with histological grade in both TCGA and Renji cohort. Knockdown VSIG4 expression in PDAC cell lines significantly inhibits its proliferation and migration. Gene set enrichment analysis (GSEA) based on TCGA database reveals that VSIG4 is negatively correlated with cytokine production. Notably, IHC shows VSIG4 is negatively correlated with infiltration of CD8^+^ T cells, and the TFs analysis explains its high expression in PDAC, providing other way to intervene VSIG4.

The reasons for the failure of immune checkpoint blockade in PDAC are multifactorial. But key contributors are the immunosuppressive tumor microenvironment (TME)—characterized by typically poor infiltration of effector T cells and PD-1^+^ T cell and prominent myeloid inflammation.[22]. VSIG4 has been reported potently inhibits the production of IL-2, the proliferation of effector CD8^+^ T cells and induces regulatory T cells (Tregs). It also inhibits the activation of pro-inflammatory macrophages by reprogramming mitochondrial pyruvate metabolism. Therefore, targeting VSIG4 in PDAC may contribute to the unlocking of checkpoint blockade in PDAC. However, since VSIG4 is characterized as a macrophage-specific molecule, neither the expression nor function of VSIG4 in any types of malignancy except gastric cancer has been reported yet. In our study, we found that high VSIG4 expression negatively correlated with anti-tumor phenotype which is consistent with previous report [9, 15]. On the one hand, high VSIG4 expression leads to increased infiltration of CD8^+^ T cells in TMA and in vitro. On the other hand, we noticed that there is a significant recruitment of myeloid cells including neutrophil and tumor-associated macrophages (TAMs) in high VSIG4 tissues and it inhibited the secretion of cytokines according to bioinformatics study. Zhang et al. found a subset of immunosuppressive P2RX1-negative neutrophils promote immune evasion and liver metastasis [23]. And promoting neutrophil polarization into anti-tumor phenotype by TGF-*β* inhibitors enhances pancreatic cancer to combined IRE and αPD1 therapy [24]. Interestingly, neutrophils may also release catalytically active neutrophil elastase (ELANE) to kill many cancer cell types while sparing non-cancer cells [25]. Therefore, it seems that further investigation of Neutrophils Atlas in PDAC by scRNA-seq is necessary. Compared to limited cognition of neutrophils in PDAC, TAMs are critical to tumorigenesis and immune evasion in PDAC [26]. Given their immunosuppressive role in the TME, targeting myeloid cells may complement immune-based strategies by removing inhibitory factors that constrain T cell responses. Therefore, it is plausible that VSIG4 may play a suppressive and inductive role in CD8^+^ cells and myeloid cells, respectively. Combination of VSIG4 and PD1 therapy may benefit PDAC patients.

Tumor-intrinsic mechanism of immune evasion is also an intense-discussed topic in recent years. Epigenetics, which was defined as changes in phenotype without changes in genotype, has achieved in early diagnosis and immunotherapies. Histone deacetylase inhibitor (HDACi), which alters chromatin accessibility and TFs binding area, inhibits VSIG4 expression in both cell lines. Wang et al. conclude that OKI-179 (isoform-selective histone deacetylase inhibitor) sensitizes lymphomas to PD1-blockade by enhancing tumor immunogenicity [27]. Combination of HDAC inhibitors with PD-1 blockade represents a promising strategy for lung cancer and melanoma treatment by increased expression of multiple T cell chemokines or PD-L1 receptor in cancer cells, macrophages and T cells [28, 29]. Combination therapies have shown a positive effect in multiple cancer. Therefore, an appropriate selection of patients who may potentially benefit from given immunotherapies is essential, and a detailed IHC-based determination of IC molecules and TIL levels should be formulated for precision treatment.

Our study preliminarily investigates biological function of VSIG4 in PDAC. There are still some limitations in our study. Relationship between myeloid cells, CD8^+^ T cells and PDAC needs further discussion, which should base on detailed subpopulation analysis by scRNA-seq to make more rational treatment schematic. Besides, inhibiting VSIG4 directly by mono-antibody or effective small molecules and combination of PD1/PDL-1 therapy in vivo is needed to evaluate immune microenvironment in future.

## Conclusions

Our results indicate that VSIG4 overexpression in PDAC is strongly related to poor prognosis. Knockdown VSIG4 in PDAC cell lines significantly inhibits proliferation and migratory ability. Besides, inhibiting VSIG4 improves immunologically “cold” TME by promoting TILs and decreasing myeloid cells infiltration, respectively. Thus, VSIG4 can serve as a promising target molecule for novel immunotherapies against PDAC. Combination of VSIG4, HDACi and first-line PD1/PDL-1 offers novel therapeutic methods for PDAC.

## Abbreviations

CRIg: Complement receptor of the immunoglobulin superfamily
EMT: Epithelial mesenchymal transition
FFPE: Formalin-fixed paraffin-embedded
GSEA: Gene set enrichment analysis
HAT: Histone acetyltransferase
HDACi: Histone deacetylase inhibitor
HPA: Human Protein Atlas
IC: Immune checkpoint
IHC: Immunohistochemistry
PDAC: Pancreatic ductal adenocarcinoma
PPI: Protein–protein interaction
TAMs: Tumor-associated macrophages
TF: Transcription factor
TMA: Tissue microarray
TME: Tumor microenvironment
Tregs: Regulatory T cells
VSIG4: V-set Ig domain-containing 4
